# Technology-enhanced learning frequency and perceived academic acceleration in AI-mediated digital learning environments: self-efficacy as an indirect pathway and learning motivation as a boundary condition

**DOI:** 10.3389/fpsyg.2026.1803874

**Published:** 2026-07-23

**Authors:** Xuezhen Chen, Zexuan Qi, Yifan Ou

**Affiliations:** 1 School of Marxism, Wenzhou Polytechnic, Wenzhou, China; 2 Xinjiang College of Science and Technology, Korla, China; 3 School of Social Sciences, University of Manchester, Manchester, United Kingdom; 4 Department of Communication Science, University of Antwerp, Antwerpen, Belgium

**Keywords:** higher education, learning motivation, perceived academic acceleration, self-efficacy, technology-enhanced learning frequency

## Abstract

AI-mediated digital learning environments have transformed how students communicate with knowledge, access learning resources, and receive algorithmically generated feedback, thereby reshaping how they perceive the pace of academic progress.This study examines perceived academic acceleration as a learning outcome distinct from general achievement, focusing on students’ self-reported sense that they move through academic tasks and course materials more quickly when supported by digital tools. A cross-sectional survey model was used to test whether technology-enhanced learning frequency was associated with perceived academic acceleration directly and indirectly through self-efficacy, and whether learning motivation conditioned the technology-acceleration association. Item-level data from 420 undergraduate students were analyzed using reliability analysis, correlation analysis, measurement-model diagnostics, ordinary least squares regression, bootstrap indirect-effect estimation, moderation analysis, and collinearity diagnostics. Results indicated that technology-enhanced learning frequency, self-efficacy, and learning motivation were positively associated with perceived academic acceleration. The indirect association through self-efficacy was supported by bootstrap confidence intervals, suggesting that students who reported more frequent technology-enhanced learning within AI-mediated learning environments also reported stronger efficacy beliefs, which in turn were associated with stronger perceived academic acceleration. The interaction between technology-enhanced learning frequency and learning motivation was positive but did not reach conventional significance. The findings should therefore be interpreted as evidence of associations among students’ technology use, efficacy beliefs, motivation, and perceived acceleration, not as causal evidence that technology use produces faster academic progress.These findings contribute to the emerging literature on AI-mediated learning and digital communication by demonstrating how technology-supported learning experiences are associated with students’ perceptions of academic progress through psychological mechanisms.The study clarifies the conceptual boundary of perceived academic acceleration and highlights the need for longitudinal and behavioral validation in future research.

## 1 Introduction

Technology-supported learning has become a routine feature of higher education. Digital platforms, online resources, intelligent learning tools, and blended instructional systems, including small private online courses (SPOCs), influence how students organize study time, access feedback, and move through course materials. These developments have encouraged researchers to examine whether technology supported learning is linked to academic performance, self-regulated learning, and student motivation (Means et al., 2013; Schmid et al., 2014). Yet the meaning of faster learning progress remains theoretically ambiguous. Students may complete tasks more quickly, perceive themselves as moving ahead, or show objective improvements in achievement. These outcomes are related but not identical. Recent research on artificial intelligence and educational technology similarly shows that digital tools increasingly reshape students’ learning routines, engagement patterns, and expectations of timely academic progress (Crompton and Burke, 2023; Godsk and Møller, 2025). Studies of generative AI and student facing learning systems further suggest that technology use should be examined not only as access to resources, but also as a perceived acceleration of learning opportunities (Chan and Hu, 2023; Yusuf et al., 2024). Blended learning research likewise indicates that technology becomes educationally meaningful when it is integrated with instructional design rather than treated as a delivery channel alone (Garrison and Kanuka, 2004). The present study focuses on perceived academic acceleration. This construct refers to students’ self-reported sense that they complete learning tasks, progress through academic materials, and move toward learning goals at a faster pace. It is not treated as objective achievement, grade improvement, or formal acceleration placement. This conceptual clarification is important because a cross sectional self-report survey can speak to perceived acceleration, but it cannot establish actual learning speed or causal academic advancement. By aligning the title, measures, results, and interpretation around perceived academic acceleration, the study avoids conflating aspiration, achievement, and task completion speed. This distinction is important because contemporary learning technologies often make progress feel faster through immediate feedback, search assistance, automated explanation, and human AI content creation, even when objective achievement is not directly measured (Khosravi et al., 2023; Molenaar, 2022). Evidence from AI assisted writing and chatbot research also indicates that students’ subjective experience of pace can differ from conventional indicators of performance (Barrot, 2023; Labadze et al., 2023).

Prior research has shown that technology use alone is not sufficient to explain learning outcomes. Students’ beliefs about their own learning capability shape how they respond to digital tools, academic challenges, and feedback opportunities (Bandura, 1997; Zimmerman, 2000). Self-efficacy is therefore a plausible psychological interface between technology enhanced learning frequency and perceived academic acceleration. Students who frequently use digital learning tools may gain more opportunities to practice, monitor progress, and obtain feedback. These experiences may strengthen efficacy beliefs, which are then associated with stronger perceptions of learning progress. Recent studies have extended this logic to AI supported and digital learning contexts, showing that self-efficacy is closely connected to online engagement, technology acceptance, and students’ perceived capacity to use learning tools productively (Getenet et al., 2024; Li et al., 2024). Research on achievement motivation and academic performance also suggests that efficacy beliefs can translate learning opportunities into more favorable academic appraisals (Li et al., 2022; Tannoubi et al., 2025). Technology enhanced learning frequency captures how often students use digital platforms, online materials, and learning tools as part of their academic work. Frequent use may increase exposure to instructional resources, feedback, examples, and practice opportunities, yet technology use becomes educationally meaningful only when students actively employ these resources to organize learning, solve academic problems, and maintain engagement. Systematic reviews of AI in online higher education indicate that technology effects vary across learning design, learner agency, and instructional support rather than following automatically from exposure alone (Ouyang et al., 2022; Limniou et al., 2022). Immersive and AI enabled systems may support academic success when they create meaningful interaction with learning tasks rather than merely increasing screen time (Sviridova et al., 2023; Ouyang and Jiao, 2021). Within this context, self-efficacy provides a theoretically important mechanism because it helps explain why similar technologies may be experienced differently by different students. This perspective is consistent with research on hybrid human AI learning technologies emphasizing learner agency and self-regulated engagement (Molenaar, 2022; Jin et al., 2023), as well as broader self-regulated learning theories that view beliefs, monitoring, and strategy use as mutually reinforcing processes (Zimmerman and Schunk, 2011).

Learning motivation may also shape how students translate technology supported opportunities into perceived progress. Motivated students are more likely to use learning tools actively rather than passively, persist when tasks are demanding, and interpret feedback as useful for improvement (Ryan and Deci, 2020). The present study therefore examines learning motivation as a boundary condition. Because the design is cross sectional, the moderation test is interpreted cautiously as an association pattern rather than evidence that motivation changes causal effects. This issue is especially relevant in AI supported higher education, where students’ motivational appraisals influence whether new tools are used strategically or only superficially (Bai and Wang, 2025; Kinskofer and Tulis, 2025). Reviews of chatbots and online learning communities further show that motivation remains central to sustained technology engagement (Huang et al., 2025; Peng and Zhang, 2024). At the same time, recent research warns that motivation may operate more strongly as a direct correlate of engagement than as a consistent boundary condition, particularly when technology use has already become routine (Bhatt and Muduli, 2024; Huang et al., 2025). The present study contributes by clarifying perceived academic acceleration as a self-reported learning pace construct, by testing self-efficacy as an indirect pathway linking technology enhanced learning frequency with perceived acceleration, and by evaluating whether learning motivation strengthens this association. The model does not claim that technology use automatically accelerates learning. Instead, it examines how technology enhanced learning frequency, efficacy beliefs, and motivation are jointly associated with students’ perceptions of academic acceleration. In doing so, the study responds to recent calls to move beyond broad claims about educational technology adoption and examine the psychological conditions under which digital resources are interpreted as academically useful (Bhatt and Muduli, 2024; Zhang et al., 2023).

From a communication perspective, AI-supported learning is increasingly understood as a form of AI-mediated communication in which students interact not only with educational content but also with algorithmically generated information, conversational agents, and intelligent feedback systems. Rather than functioning solely as instructional technologies, AI-enabled platforms reshape how learners access, interpret, and negotiate information through continuous human–AI interaction. Consequently, learning experiences in AI-mediated environments can be conceptualized as communication processes in which psychological responses, such as self-efficacy, influence how students evaluate their academic progress.

## 2 Literature review and hypotheses

Technology-enhanced learning frequency captures how often students use digital platforms, online materials, and learning tools as part of their academic work. Frequent use may increase exposure to instructional resources, feedback, examples, and practice opportunities. However, technology use is only educationally meaningful when students use it to organize learning, solve academic problems, and maintain engagement. This distinction helps explain why technology-enhanced learning frequency should be associated with perceived academic acceleration but should not be interpreted as equivalent to objective achievement. Systematic reviews of AI in online higher education indicate that technology effects vary across learning design, learner agency, and instructional support rather than following automatically from exposure alone (Ouyang et al., 2022; Limniou et al., 2022). Immersive and AI-enabled systems may support academic success when they create meaningful interaction with learning tasks rather than merely increasing screen time (Sviridova et al., 2023; Ouyang and Jiao, 2021).

*H1*. Technology-enhanced learning frequency is positively associated with perceived academic acceleration.

Self-efficacy refers to students’ beliefs that they can organize and execute actions required for academic success. In technology-supported learning settings, efficacy beliefs may be strengthened when students experience successful task completion, receive timely feedback, and learn to navigate digital learning resources. Students with stronger self-efficacy may also interpret accelerated progress as manageable rather than overwhelming. In digital contexts, this pathway is plausible because confident students are more likely to interpret technology-related challenges as manageable and to persist when tools require adjustment or self-directed problem solving (Bergdahl and Sjöberg, 2025; Jin et al., 2023).

*H2*. Technology-enhanced learning frequency is positively associated with self-efficacy.

*H3*. Self-efficacy is positively associated with perceived academic acceleration after technology-enhanced learning frequency is controlled.

The indirect pathway through self-efficacy is theoretically important because it shifts attention away from a simple technology-use explanation. If technology-enhanced learning frequency is associated with perceived academic acceleration partly through self-efficacy, then psychological beliefs help explain why similar learning technologies may be experienced differently by different students. This interpretation is also consistent with work on hybrid human-AI learning technologies, which emphasizes that learner agency and self-regulated engagement mediate the value of intelligent support (Molenaar, 2022; Jin et al., 2023). This view is consistent with self-regulated learning research that treats learners’ beliefs, monitoring, and strategy use as mutually reinforcing processes rather than isolated traits (Zimmerman and Schunk, 2011).

*H4*. Technology-enhanced learning frequency has a positive indirect association with perceived academic acceleration through self-efficacy.

Learning motivation reflects students’ willingness to invest effort, persist in academic work, and seek improvement. Motivation may shape whether frequent technology use becomes an active learning resource or merely a convenient access channel. The moderation hypothesis is therefore examined as a conditional association rather than a causal claim. However, recent research also warns that motivation may operate more strongly as a direct correlate of engagement than as a consistent boundary condition, especially when students’ technology use is already routine (Bhatt and Muduli, 2024; Huang et al., 2025).

*H5*. Learning motivation positively moderates the association between technology-enhanced learning frequency and perceived academic acceleration.

## 3 Methods

### 3.1 Participants and procedure

The study used a cross-sectional survey design with undergraduate students in technology-supported learning contexts. Data were collected via Wenjuanxing, an online survey platform widely used in Chinese academic research, across four universities in southwest China. The survey link was distributed through university communication channels and student networks. Because participation was voluntary and the survey used an open-link distribution format, the total number of students who received or viewed the invitation could not be determined, and a conventional response rate could not be calculated. A total of 445 responses were received. Twenty-five were excluded because of excessive missing values (more than 20% of items missing), clearly patterned answers, implausibly fast completion times (below one-third of the median completion time), or straightlining on any single construct scale. The final analytic sample consisted of 420 students aged 18 to 26 years. Participants represented multiple study years and academic fields, including education, engineering, business, humanities, and health sciences. Inclusion criteria required that participants were currently enrolled undergraduate students with experience using digital or online tools for learning tasks. Participation was voluntary and anonymous. The questionnaire was administered in both Chinese and English; participants completed the Chinese version, and the English item wordings presented in this report correspond directly to the Chinese source items, with both versions developed in parallel to ensure conceptual equivalence. The questionnaire assessed technology-enhanced learning frequency, self-efficacy, learning motivation, perceived academic acceleration, learning pace pressure, and demographic information. All focal constructs were measured using four-item scales with responses from 1 (strongly disagree) to 7 (strongly agree).

### 3.2 Measures

Technology-enhanced learning frequency was assessed using items adapted from prior studies examining students’ engagement with digital learning environments and online learning resources (Sun and Rueda, 2012; Broadbent and Poon, 2015). Self-efficacy was measured using items reflecting students’ beliefs in their capability to successfully perform academic tasks, consistent with social cognitive theory (Bandura, 1997; Zimmerman, 2000). Learning motivation was assessed using items capturing effort, persistence, and the desire to improve academic performance, drawing on self-determination theory and motivational research (Deci and Ryan, 2000; Ryan and Deci, 2020). Because perceived academic acceleration represents a relatively unexplored construct, four items were developed specifically for this study based on the conceptual definition proposed in the literature review. Learning pace pressure was measured using items reflecting perceived pressure to keep up with demanding academic expectations (Pintrich, 2000; Schunk and DiBenedetto, 2020). All measures employed a seven-point Likert response format ranging from strongly disagree to strongly agree.

### 3.3 Data analysis

Data analyses were conducted using Python 3.12.13 with NumPy, Pandas, and OpenPyXL. Internal consistency was assessed using Cronbach’s alpha. Convergent validity was evaluated through composite reliability (CR) and average variance extracted (AVE), whereas discriminant validity was examined using heterotrait–monotrait (HTMT) ratios. Confirmatory factor analysis (CFA) was conducted to evaluate the adequacy of the proposed five-factor measurement model. Hypotheses were tested using ordinary least squares regression. Indirect associations were estimated using 5,000 bootstrap resamples, and moderation effects were examined using standardized product terms. Collinearity diagnostics were calculated for all predictor variables included in the regression models. Reliability and validity assessments followed established recommendations for multivariate analysis and structural equation modeling (Hair et al., 2019; Kline, 2023). HTMT values were interpreted according to established guidelines for discriminant validity (Henseler et al., 2015). Indirect effects were evaluated using contemporary mediation and conditional-process procedures while avoiding causal interpretation because of the cross-sectional design (Hayes, 2022). Potential common method bias was considered in accordance with recommendations for self-report research (Podsakoff et al., 2012). All analyses were implemented in Python, and model estimation procedures followed widely adopted open-source structural equation modeling conventions (Rosseel, 2012).

## 4 Results

As shown in Table 1, the mean scores of the focal constructs were generally above the scale midpoint, indicating that participants reported relatively frequent engagement with technology-supported learning activities, moderate levels of academic self-efficacy and learning motivation, and a moderately positive perception of academic acceleration. Learning motivation displayed the highest mean score, whereas learning pace pressure was comparatively lower. The distribution of responses showed adequate variability, and the observed score ranges suggested no substantial floor or ceiling effects. These descriptive results provide preliminary support for the suitability of the data for subsequent hypothesis testing.

**Table 1:** Descriptive statistics of study variables.

Variable	*N*	Mean	SD	Min	Max
Technology-enhanced learning frequency	420	4.430	0.860	2.250	7.000
Self-efficacy	420	4.399	0.900	1.750	7.000
Learning motivation	420	4.567	0.862	1.250	6.750
Perceived academic acceleration	420	4.167	0.892	1.750	7.000
Learning pace pressure	420	3.714	0.843	1.500	6.250
Age	420	20.750	1.701	18.000	26.000
Female (%)	420	61.429			

Means differed across constructs, reducing concern that the results reflect mechanical scale duplication. Perceived academic acceleration was measured as a self-reported learning-pace perception rather than objective achievement.

Table 2 presents the bivariate correlations among the study variables. Technology-enhanced learning frequency, self-efficacy, learning motivation, and perceived academic acceleration were positively associated with one another, with correlation coefficients ranging from 0.168 to 0.594. The strongest association was observed between self-efficacy and perceived academic acceleration (*r* = 0.594, *p* < 0.001). Learning pace pressure showed relatively weak relationships with the focal constructs and was not significantly associated with either self-efficacy or perceived academic acceleration. The magnitude of the correlations suggests that the variables were related while remaining sufficiently distinct to justify subsequent multivariate analyses.

**Table 2:** Correlations.

Variable	Technology-enhanced learning frequency	Self-efficacy	Learning motivation	Perceived academic acceleration	Learning pace pressure
Technology-enhanced learning frequency	1.000				
Self-efficacy	0.447***	1.000			
Learning motivation	0.168***	0.290***	1.000		
Perceived academic acceleration	0.500***	0.594***	0.331***	1.000	
Learning pace pressure	0.227***	0.011	0.118*	0.053	1.000

Correlations show the expected positive pattern among technology-enhanced learning frequency, self-efficacy, learning motivation, and perceived academic acceleration. The magnitudes are moderate rather than redundant. *p < 0.05, **p < 0.01, and ***p < 0.001.

Table 3 shows that all constructs demonstrated satisfactory internal consistency and convergent validity. Cronbach’s alpha values ranged from 0.856 to 0.888, exceeding the commonly recommended threshold of 0.70. Composite reliability values ranged from 0.903 to 0.923, indicating strong construct reliability. In addition, all average variance extracted values were above 0.50, suggesting that the indicators explained a substantial proportion of variance in their respective constructs. These results support the reliability and convergent validity of the measurement model.

**Table 3:** Reliability.

Construct	Items	Cronbach’s *α*	AVE	CR
Technology-enhanced learning frequency	4	0.868	0.716	0.910
Self-efficacy	4	0.888	0.749	0.923
Learning motivation	4	0.872	0.723	0.913
Perceived academic acceleration	4	0.885	0.743	0.920
Learning pace pressure	4	0.856	0.699	0.903

All scales exceeded common reliability thresholds. CR values are higher than or comparable to alpha values, and AVE values exceed 0.50, supporting convergent validity.

Table 4 presents the standardized loadings for all measurement items. All loadings exceeded 0.80, ranging from 0.816 to 0.873, indicating strong associations between the indicators and their respective constructs. The results provide additional support for the convergent validity of the measurement model and suggest that the items adequately captured the intended latent constructs.

**Table 4:** Item loadings.

Construct	Item	Item wording	Standardized loading
Technology-enhanced learning frequency	Item 1	I frequently use digital platforms to support my learning tasks.	0.857
Technology-enhanced learning frequency	Item 2	Technology-supported tools are part of my regular study routine.	0.816
Technology-enhanced learning frequency	Item 3	I often rely on online learning resources to complete academic work.	0.859
Technology-enhanced learning frequency	Item 4	Digital learning tools help me move through learning materials efficiently.	0.852
Self-efficacy	Item 1	I am confident that I can master difficult learning tasks.	0.867
Self-efficacy	Item 2	When I encounter academic difficulties, I believe I can find effective solutions.	0.854
Self-efficacy	Item 3	I can organize my learning effectively even when tasks are challenging.	0.868
Self-efficacy	Item 4	I believe I can achieve strong academic progress through my own effort.	0.872
Learning motivation	Item 1	I am motivated to improve my academic performance.	0.867
Learning motivation	Item 2	I put effort into learning because academic progress matters to me.	0.848
Learning motivation	Item 3	I am willing to persist when learning tasks become difficult.	0.849
Learning motivation	Item 4	I actively seek opportunities to improve my learning outcomes.	0.838
Perceived academic acceleration	Item 1	I usually complete learning tasks faster than expected.	0.869
Perceived academic acceleration	Item 2	I can progress through course materials at an accelerated pace.	0.860
Perceived academic acceleration	Item 3	My learning performance improves quickly when I use available learning resources.	0.846
Perceived academic acceleration	Item 4	I often move ahead of the expected learning schedule.	0.873
Learning pace pressure	Item 1	I often feel pressure to keep up with a fast learning pace.	0.844
Learning pace pressure	Item 2	Academic tasks sometimes require me to progress faster than I prefer.	0.837
Learning pace pressure	Item 3	I feel that learning expectations have become increasingly accelerated.	0.827
Learning pace pressure	Item 4	I often need to adapt quickly to changing learning requirements.	0.838

All standardized loadings exceeded 0.80, indicating strong relationships between the items and their intended constructs.

Table 5 reports the HTMT ratios among the study constructs. All values were substantially below the recommended threshold of 0.85, ranging from 0.042 to 0.671. The highest HTMT value was observed between self-efficacy and perceived academic acceleration (HTMT = 0.671), which remained well below the critical cutoff. These findings provide evidence of satisfactory discriminant validity and indicate that the constructs represent related but empirically distinct concepts.

**Table 5:** HTMT ratios among the study constructs.

Construct	Technology-enhanced learning frequency	Self-efficacy	Learning motivation	Perceived academic acceleration	Learning pace pressure
Technology-enhanced learning frequency	1.000				
Self-efficacy	0.510	1.000			
Learning motivation	0.193	0.331	1.000		
Perceived academic acceleration	0.570	0.671	0.378	1.000	
Learning pace pressure	0.264	0.042	0.137	0.060	1.000

As shown in Table 6, the five-factor measurement model demonstrated satisfactory fit to the data. The values of CFI (0.941) and TLI (0.938) approached or exceeded commonly recommended benchmarks, while RMSEA (0.059) and SRMR (0.044) remained within acceptable ranges. Together with the reliability, convergent validity, and discriminant validity results reported previously, these findings support the adequacy of the measurement model and the empirical distinctiveness of the study constructs.

**Table 6:** Measurement model.

Model	*χ* ^2^	df	*χ*^2^/df	CFI	TLI	RMSEA	SRMR
Five-factor measurement model	445.962	180	2.478	0.941	0.938	0.059	0.044

Table 7 presents the regression results predicting perceived academic acceleration. Technology-enhanced learning frequency was positively associated with perceived academic acceleration (*B* = 0.307, *p* < 0.001), supporting H1. Self-efficacy also showed a positive association with perceived academic acceleration after controlling for the other predictors (*B* = 0.410, *p* < 0.001), supporting H3. Learning motivation was positively associated with perceived academic acceleration (*B* = 0.172, *p* < 0.001). In contrast, learning pace pressure was not significantly associated with perceived academic acceleration (*B* = −0.041, *p* = 0.305). Among the predictors, self-efficacy exhibited the strongest association with perceived academic acceleration, suggesting that students’ confidence in their academic capabilities may play a particularly important role in shaping perceptions of learning progress (Table 8).

**Table 7:** Regression results predicting perceived academic acceleration.

Variable	*B*	SE	*t*	*p*	Sig.
Constant	0.370	0.256	1.447	*p* = 0.148	n.s.
Technology-enhanced learning frequency	0.307	0.044	7.029	<0.001	***
Self-efficacy	0.410	0.042	9.763	<0.001	***
Learning motivation	0.172	0.040	4.316	<0.001	***
Learning pace pressure	−0.041	0.040	−1.026	*p* = 0.305	n.s.

***indicates that the reported coefficient is statistically significant at the *p* < 0.001 level.

**Table 8:** Overall fit of the regression model.

Outcome	*R* ^2^	Adjusted *R*^2^	*F*	df1	df2
Perceived academic acceleration	0.447	0.442	83.872	4	415

The overall regression model was statistically significant, *F*(4, 415) = 83.87, and explained 44.7% of the variance in perceived academic acceleration (*R*^2^ = 0.447, Adjusted *R*^2^ = 0.442). The small difference between *R*^2^ and adjusted *R*^2^ suggests that the model was not substantially affected by overfitting. Overall, the results indicate that technology-enhanced learning frequency, self-efficacy, learning motivation, and learning pace pressure jointly accounted for a substantial proportion of individual differences in perceived academic acceleration.

Table 9 presents the bootstrap mediation results. Technology-enhanced learning frequency was positively associated with perceived academic acceleration through both direct and indirect pathways. The indirect effect through self-efficacy was significant (Effect = 0.215, SE = 0.028, 95% CI [0.162, 0.271]), as the confidence interval did not include zero. The direct effect remained significant after accounting for self-efficacy (Effect = 0.304, SE = 0.043, 95% CI [0.220, 0.389]), indicating partial mediation. Approximately 41.4% of the total association between technology-enhanced learning frequency and perceived academic acceleration was transmitted through self-efficacy, providing support for H4.

**Table 9:** Bootstrap mediation analysis of self-efficacy.

Path	Effect	SE	95% CI	Sig.
Total effect (c)	0.519	0.044	[0.433, 0.605]	***
Direct effect (c’)	0.304	0.043	[0.220, 0.389]	***
Indirect effect via self-efficacy	0.215	0.028	[0.162, 0.271]	Supported

Bootstrap confidence intervals were generated using 5,000 resamples. An indirect effect was considered significant when the confidence interval did not include zero. ***indicates that the reported coefficient is statistically significant at the *p* < 0.001 level.

Table 10 presents the moderation results. Technology-enhanced learning frequency remained positively associated with perceived academic acceleration (*B* = 0.298, *p* < 0.001), and learning motivation also showed a significant positive association (*B* = 0.166, *p* < 0.001). However, the interaction term between technology-enhanced learning frequency and learning motivation was not statistically significant (*B* = 0.054, *p* = 0.106). These findings do not support H5 and suggest that the positive association between technology-enhanced learning frequency and perceived academic acceleration did not vary significantly across different levels of learning motivation. Self-efficacy remained a significant positive predictor, whereas learning pace pressure was not significantly associated with perceived academic acceleration.

**Table 10:** Moderation model predicting perceived academic acceleration.

Variable	*B*	SE	*t*	*p*	Sig.
Constant	−1.874	0.285	−6.579	<0.001	***
Technology-enhanced learning frequency	0.298	0.042	7.084	<0.001	***
Learning motivation	0.166	0.038	4.329	<0.001	***
TELF x learning motivation	0.054	0.033	1.617	*p* = 0.106	n.s.
Self-efficacy	0.459	0.047	9.736	<0.001	***
Learning pace pressure	−0.041	0.045	−0.910	*p* = 0.363	n.s.

The interaction term between technology-enhanced learning frequency and learning motivation was included to test the moderation hypothesis. ***indicates that the reported coefficient is statistically significant at the *p* < 0.001 level.

Table 11 presents the standardized regression coefficients. Self-efficacy exhibited the strongest positive association with perceived academic acceleration (*β* = 0.414, *p* < 0.001), followed by technology-enhanced learning frequency (*β* = 0.296, *p* < 0.001) and learning motivation (*β* = 0.166, *p* < 0.001). Learning pace pressure was not significantly associated with perceived academic acceleration (*β* = −0.039, *p* = 0.305). The standardized estimates suggest that self-efficacy was the most influential predictor in the model, highlighting the importance of students’ efficacy beliefs in shaping perceptions of academic progress.

**Table 11:** Standardized regression coefficients.

Variable	*B*	SE	*t*	*p*	Sig.
Constant	0.000	0.037	0.000	*p* = 1.000	n.s.
Technology-enhanced learning frequency (std)	0.296	0.042	7.029	<0.001	***
Self-efficacy (std)	0.414	0.042	9.763	<0.001	***
Learning motivation (std)	0.166	0.038	4.316	<0.001	***
Learning pace pressure (std)	−0.039	0.038	−1.026	*p* = 0.305	n.s.

Standardized coefficients allow comparison of predictor magnitudes. Self-efficacy showed the largest standardized association with perceived academic acceleration. ***indicates that the reported coefficient is statistically significant at the *p* < 0.001 level.

Table 12 reports the collinearity diagnostics for the predictor variables. All variance inflation factor values were close to 1 and well below commonly accepted thresholds, while tolerance values remained high. These results indicate that multicollinearity was not a concern in the regression analyses and suggest that the estimated coefficients can be interpreted with confidence.

**Table 12:** Collinearity diagnostics.

Variable	VIF	Tolerance	Assessment
Technology-enhanced learning frequency	1.332	0.751	Acceptable
Self-efficacy	1.351	0.740	Acceptable
Learning motivation	1.108	0.902	Acceptable
Learning pace pressure	1.079	0.926	Acceptable

Table 13 presents the robustness analysis. The coefficients for technology-enhanced learning frequency and self-efficacy remained virtually unchanged after age was added as an additional control variable. The explained variance also remained identical across specifications. These findings suggest that the main results were robust to the inclusion of age and were not driven by age-related differences within the sample.

**Table 13:** Robustness.

Specification	TELF B	Self-efficacy B	*R* ^2^	Conclusion
Main model	0.307	0.410	0.447	Main associations retained
Age-adjusted model	0.307	0.411	0.447	Stable after age control

The main associations were stable when age was added as an additional control variable.

## 5 Discussion

This study examined how technology-enhanced learning frequency, self-efficacy, and learning motivation are associated with perceived academic acceleration. The results supported a positive association between technology-enhanced learning frequency and perceived academic acceleration, while self-efficacy was positively associated with perceived acceleration and accounted for a substantial indirect pathway linking technology-enhanced learning frequency with perceived academic acceleration. These findings suggest that students’ beliefs about their academic capability play an important role in how technology-supported learning is experienced and interpreted. At the same time, the results should not be read as evidence that technology use causes faster learning or that self-efficacy causes academic acceleration. Because the study relied on a cross-sectional design, temporal ordering cannot be established. A more cautious interpretation is that students who report more frequent engagement with technology-supported learning tools also tend to report stronger efficacy beliefs and stronger perceptions of academic progress. This interpretation is consistent with methodological guidance indicating that mediation estimates derived from self-report survey data should be viewed as indirect associations unless stronger temporal or experimental evidence is available (Hayes, 2022; Podsakoff et al., 2012). Future research should therefore employ longitudinal designs that measure technology engagement, efficacy development, and learning outcomes at different time points to clarify the processes through which technology-supported learning becomes associated with perceived academic acceleration.

The non-significant moderation result is also theoretically informative. Although learning motivation was positively associated with perceived academic acceleration, it did not significantly alter the relationship between technology-enhanced learning frequency and perceived acceleration. One possible explanation is that the motivation measure captured general academic effort and persistence rather than technology-specific motivational engagement. Another possibility is that a cross-sectional survey cannot adequately capture moment-to-moment variations in how students interact with digital learning tools under different academic demands. Recent studies similarly suggest that motivational beliefs may contribute to overall engagement without necessarily strengthening specific relationships between technology use and perceived outcomes in every educational context (Kinskofer and Tulis, 2025; Peng and Zhang, 2024). From a practical perspective, the findings indicate that educational technology should not be evaluated solely in terms of access, availability, or frequency of use. Institutions and instructors should consider whether technology-supported learning environments enhance students’ confidence, provide meaningful feedback, and support clear perceptions of progress. Digital tools that increase efficiency without strengthening efficacy beliefs or self-regulatory capacity may create perceptions of acceleration without producing durable learning benefits. This interpretation aligns with recent evidence showing that digital literacy, technology attitudes, and instructional design influence how students engage with educational technologies and derive value from them (Getenet et al., 2024; Godsk and Møller, 2025).

### 5.1 Limitations and future research

Several limitations should be acknowledged. The design was cross-sectional and relied on self-report measures, which limits causal inference and raises the possibility of common method bias despite the observed discriminant validity among the constructs. Perceived academic acceleration was conceptualized as a self-reported learning pace construct and was measured using items developed for the present study. Although the scale demonstrated satisfactory internal consistency and construct validity in the current sample, additional validation is needed. Future studies should examine its content validity, test–retest reliability, and external validity across independent samples and educational contexts. Furthermore, perceived academic acceleration was not validated against objective indicators such as academic grades, task completion time, learning analytics, or instructor assessments. The sample consisted of undergraduate students in technology-supported learning settings and may not generalize to younger learners, adult professional learners, postgraduate students, or fully online degree programs. Future research should employ longitudinal and multi-wave designs to establish temporal ordering among technology use, self-efficacy, motivation, and perceived acceleration. Researchers should also combine subjective perceptions with objective indicators such as course performance, learning analytics, completion time, and platform log-file traces, as recommended in broader AI in education and technology enhanced learning research (Ouyang et al., 2022; Zhang et al., 2023). In addition, future studies may explore whether the observed relationships vary across different forms of educational technology, levels of digital literacy, and AI supported learning environments.

## 6 Conclusion

This study clarifies perceived academic acceleration as a self-reported learning-pace construct and examines its associations with technology-enhanced learning frequency, self-efficacy, and learning motivation. The results suggest that technology-enhanced learning frequency is positively associated with perceived academic acceleration and that self-efficacy accounts for part of this association. Learning motivation showed a positive direct association but did not significantly moderate the technology-acceleration link. The findings support a cautious psychological interpretation: technology-supported learning is most meaningful when it is connected with students’ confidence, motivation, and perceptions of manageable progress.

Beyond contributing to educational technology research, this study also contributes to the emerging literature on AI-mediated learning and digital communication in higher education. As AI increasingly functions as an interactive communication partner rather than merely an instructional tool, understanding students’ psychological responses to AI-mediated learning experiences becomes increasingly important. The findings suggest that technology-supported learning becomes educationally meaningful not simply because digital tools are available, but because students interpret AI-generated feedback and learning experiences through psychological mechanisms such as self-efficacy. This perspective extends current discussions of AI-supported learning by highlighting the communication processes through which intelligent technologies become associated with students’ perceived academic progress.

## Data Availability

The raw data supporting the conclusions of this article will be made available by the authors, without undue reservation.
